# Optimal composition of movement behaviours for healthy weight across the lifespan: a one-stage individual participant data meta-analysis of 11 studies

**DOI:** 10.21203/rs.3.rs-10064313/v1

**Published:** 2026-07-01

**Authors:** Aleš Gába, Željko Pedišić, Timothy B Hartwig, Paulína Ševčíková, Taren Sanders, Jan Dygrýn, Ondřej Vencálek, Devan Antczak, James H Conigrave, Phillip Parker, Borja del Pozo Cruz, Stuart J Fairclough, Shona Halson, Michael Noetel, Manuel Ávila-García, Veronica Cabanas-Sánchez, Iván Cavero-Redondo, Rachel G Curtis, Bruno GG da Costa, Jesus del Pozo-Cruz, Antonio García-Hermoso, Angus A Leahy, David R Lubans, Carol A Maher, David Martínez-Gómez, Kim Meredith-Jones, Manasa Shanta Näsholm, Andrés Redondo-Tébar, Séverine Sabia, Kelly S Silva, Emilio Villa-González, Chris Lonsdale

**Affiliations:** Palacky University Olomouc: Univerzita Palackeho v Olomouci; University of Hong Kong Yu Chun Keung Medical Library: The University of Hong Kong Li Ka Shing Faculty of Medicine; Australian Catholic University; Palacký University Olomouc; Australian Catholic University; Palacký University Olomouc; Palacký University Olomouc; Pentara Corporation; La Trobe University; Australian Catholic University; Universidad Europea de Madrid; Edge Hill University; Australian Catholic University; University of Queensland; University of Granada; Universidad Autónoma de Madrid; Universidad de Castilla-La Mancha; Adelaide University; McGill University; Universidad de Sevilla; Universidad Pública de Navarra; University of Newcastle; University of Newcastle; Adelaide University; Universidad Autónoma de Madrid; University of Otago; Karolinska Institutet; Universidad de Castilla-La Mancha; Université Paris Cité; Universidade Federal de Santa Catarina; University of Granada; Australian Catholic University

## Abstract

**Background:**

Identifying the optimal balance between daily movement behaviours might be valuable for developing effective strategies to achieve a healthy weight. However, there remains a significant research gap in understanding how the optimal movement behaviour compositions vary between different populations and contexts.

**Objective:**

To determine the optimal movement behaviour composition for healthy weight across the lifespan.

**Methods:**

Following PRISMA guidelines, this one-stage individual participant data meta-analysis harmonised data from observational or intervention studies that collected 24-hour movement-behaviour data using research-grade accelerometers and assessed body mass index (BMI) and/or waist circumference. Reprocessed raw accelerometer-derived data provided estimates of sleep duration and time spent in sedentary behaviour (SB), light physical activity (LPA), and moderate-to-vigorous physical activity (MVPA). Healthy weight was defined as body mass index and waist circumference within the “healthy” ranges. The optimal daily movement behaviour composition was defined as the compositional mean of movement behaviour compositions associated with healthy weight.

**Results:**

Harmonised data from 9,818 participants across 11 studies conducted in Australia, Brazil, Chile, Czechia, New Zealand, Spain and the UK were included. For children, the optimal daily composition of movement behaviours for “healthy” BMI included 9.1h of sleep, 9.9h of SB, 3.8h of LPA, and 1.2h of MVPA. For adolescents, it included 7.4h of sleep, 12.6h of SB, 3.5h of LPA, and 0.6h of MVPA. The optimal daily composition of movement behaviours for healthy weight, calculated as a pooled estimate for “healthy” BMI and “healthy” waist circumference, consisted of 7.9h of sleep, 12.1h of SB, 2.4h of LPA, and 1.6h of MVPA for adults and 7.9h of sleep, 12.6h of SB, 2.3h of LPA, and 1.2h of MVPA for older adults.

**Conclusions:**

Movement behaviour composition is associated with weight-related outcomes in all age groups. Our findings could be used to optimise movement behaviours within weight management strategies across the lifespan, and to inform the development of future movement behaviour guidelines.

## 1 Introduction

In a 24-hour day, the time spent in physical activity (PA), sedentary behaviour (SB), and sleep forms a specific time-use composition known as “movement behaviour composition”. Until approximately 10 years ago, these behaviours were typically studied individually, largely overlooking their co-dependency [1]. However, in recent years, an increasing number of studies have started considering movement behaviours integratively, most commonly by categorising participants based on adherence to 24-hour movement guidelines or by using different types of isotemporal substitution analyses [2, 3].

In a seminal paper, Tremblay and colleagues suggested that an appropriate balance between physical activity, physical inactivity, and sleep is required for good health [4]. Several theoretical models were subsequently developed to provide methodological frameworks for future studies on optimal balance between movement behaviours [1, 5–8]. However, only a few empirical studies have attempted to determine the optimal composition of movement behaviours. Among these, pioneering work done by Dumuid and colleagues [9] explored optimal composition of movement behaviours for skeletal health among children. The authors proposed a statistical procedure for identifying the optimal composition of movement behaviours, which was later used in several other studies, analysing a range of health outcomes in various population groups [10–19]. These studies examined cardiometabolic, musculoskeletal, psychosocial and mental health, as well as cognitive and executive functioning and motor and physical fitness outcomes among preschoolers, children and adolescents [10–16]. In adult populations, outcomes included cardiometabolic risk and glycaemic control among middle-aged and older adults,[17, 18] and back pain among eldercare workers [19].

Although the World Health Organization (WHO) formally recognised the global epidemic of obesity as a key public health issue more than three decades ago [20], the prevalence of obesity continues to increase worldwide. Recent estimates suggest that approximately 2.6 billion people are currently living with overweight or obesity, and projections indicate that by 2050 more than one third of children and adolescents and over half of adults will be affected [21, 22]. Given the substantial and growing economic burden associated with overweight and obesity [23], addressing this epidemic remains a global public health priority. Previous findings suggest that reallocations of time from physical activity to other movement behaviours, especially to SB, are associated with unfavourable obesity outcomes and vice versa [24, 25]. According to the *Sweet-Spot Hypothesis* [7], the association between movement-behaviour composition and obesity can be explained by the biological theory of *allostasis* [26]. A long-term imbalance between movement behaviours may be associated with a cumulative physiological and psychological burden, known as *allostatic load* [27, 28], which can lead to adverse adaptations [28]. Although its aetiology is multifactorial, obesity is considered as one of the unhealthy allostatic adaptations linked to an imbalance between movement behaviors [29]. Therefore, it is important to identify the optimal composition of movement behaviours for healthy weight, as a starting point for developing causal hypotheses and informing intervention design.

Previous studies provided insights into the optimal composition of movement behaviours for healthy weight among children in Australia [10, 11], children, adolescents and older adults in Czechia [14, 18], and middle-aged and older adults in the Netherlands [17]. However, generalizability of their findings to other countries and world regions is limited, and the reproducibility of their results needs to be established in diverse cohorts. In addition, making comparisons between different age groups based on previous findings is challenging, due to differences in data processing methods across the studies. No previous study has attempted to harmonise data from different studies and countries, to determine the optimal movement behaviour composition for healthy weight. Also, no previous study on this topic included participants from all age groups. Therefore, the aim of this study was to determine the optimal movement behaviour composition for healthy weight across the lifespan, by conducting a one-stage individual participant data (IPD) meta-analysis of 11 studies from 7 countries.

## 2 Methods

The data used in the current study were pooled and harmonised as part of the project “The relationship between sleep and physical activity across the lifespan”, by following a registered protocol [30]. Although the project was primarily focused on the association between sleep and physical activity, the pooled dataset also included weight status indicators, which enabled us to conduct the current analyses. We adhered to the methodological framework outlined in the original protocol, while also conducting additional analyses that were necessary to achieve our current research objectives. In this study, we followed the Preferred Reporting Items for Systematic reviews and Meta-Analyses (PRISMA) guidelines for IPD meta-analyses [31], ensuring standardised and rigorous reporting. Herein, we provide a summary of the methods used in our study, while more details can be found elsewhere [32].

### 2.1 Search strategy and inclusion criteria

We used a multilevel search strategy to identify eligible datasets, including: 1) backward citation tracking by reviewing reference lists of the key systematic reviews related to the primary objective of the project [30]; 2) reviewing reference lists of relevant studies identified in the previous step; 3) searching for unpublished research and grey literature; 4) identifying consortia with 24-hour accelerometer data on movement behaviours; and 5) directly contacting researchers to help us identify other relevant data sources.

Studies were eligible for inclusion, if they: 1) collected 24-hour data on movement behaviours using research-grade accelerometers; 2) collected data on indicators of weight status; 3) included ≥ 400 participants aged four years or older; and 4) received approval from an institutional research ethics committee. Cross-sectional, longitudinal, and interventional study designs were considered. However, we used only baseline data from intervention studies to ensure that the intervention component does not influence our findings.

### 2.2 Processing accelerometer data

After identifying eligible studies, we invited their corresponding authors to provide de-identified individual raw accelerometer data and additional study-level data (e.g. accelerometer type and wear-time protocol) via a secure file-sharing system. For harmonisation purposes, the raw accelerometer data were reprocessed at the study coordinating centre or by contributing authors and data custodians, depending on the data-sharing policies of their institutions.

Wrist-worn accelerometer data were reprocessed using the *sleepIPD* R package version 0.2.2,[33] built upon the *GGIR* package [34]. This ensured standardised processing of data (e.g., auto-calibration using local gravity as a reference) collected in various parts of the world and in various formats, while minimizing the risk of methodological variations affecting the results. *GGIR*'s default algorithms were used for sleep period detection [35], while the Euclidean Norm Minus One thresholds were applied to estimate time spent in SB, light-intensity physical activity (LPA), and moderate-to-vigorous physical activity (MVPA) [36–38]. Specific thresholds, according to participant age and accelerometer type, are detailed in the *sleepIPD* documentation [33]. Various data processing protocols can be used to estimate the amounts of time spent in sleep, SB, LPA, and MVPA from raw acceleration. Given that the estimates may differ substantially depending on the selected protocol, we also calculated the average acceleration (expressed in the units of Earth’s gravitational acceleration, m*g*) and the intensity gradient, as a measure of uniformity of distribution of time across the activity intensity spectrum [39]. Participants included in the analytic dataset had to have a minimum wear time of three valid weekdays and one valid weekend day. A day was considered as valid, if it had a wear time of 540 to 1,080 minutes during wake time, sleep duration (defined as the sleep period time window) of 320 to 900 minutes, and a combined total of at least 960 minutes.

### 2.3 Identifying healthy weight

A healthy weight was defined by considering body mass index (BMI) and waist circumference within the age- and sex-specific “healthy” ranges. For adults and older adults, the categorisation according to waist circumference was done using the BMI-specific thresholds. This approach is consistent with the current recommendations, as combining BMI with additional anthropometric measures improves the identification of weight status and reduces misclassification at the individual level [40]. As an indicator of healthy weight, we considered a WHO BMI *z*-score between − 2 and + 1 among children (5–12 years of age) and adolescents (13–17 years of age), and BMI between 18.5 and 24.9 kg/m^2^ among adults (18–64 years of age), and older adults (≥ 65 years of age). Waist circumference below the 85th sex- and age-specific percentile [41], the most frequently used threshold for overweight [42], was considered as an indicator of healthy weight among children and adolescents. For adults and older adults, sex- and BMI-specific waist circumference thresholds were applied [43].

### 2.4 Statistical analysis

We calculated absolute and relative frequencies for categorical variables; arithmetic means and standard deviations for non-compositional continuous variables; and compositional means and total variance for compositional data. The estimation of optimal composition was performed in three steps, separately for children (5–12 years), adolescents (13–17 years), adults (18–64 years), and older adults (65 years and older), and further stratified by sex and socioeconomic status (SES).

First, multi-level multivariate regression models with random intercepts for study identification number were employed, to determine the associations between movement behaviour composition (consisting of the times spent in sleep, SB, LPA, and MVPA) as fixed effects and an indicator of weight status as the outcome variable. To account for the compositional nature of movement behaviour data, the raw composition was expressed as pivot coordinates [44], which were then included in mixed models adjusted for age, sex, and SES. We employed Type II analysis of variance to determine whether the movement-behaviour composition is associated with BMI and waist circumference. Given that 795 missing values for SES were imputed using the iterative robust model-based imputation technique [45], a sensitivity analysis was conducted to determine whether this procedure affected the results of regression analysis. As the inclusion of imputed SES led to the *F*-statistic exceeding the critical threshold in the model with waist circumference among children (Table S2), we excluded participants with missing data from this particular analysis. A detailed description of the identification and selection of potential confounders, as well as the strategy for missing data imputation, is provided in the Supplementary Files.

Second, we utilised all movement behaviour compositions available in the dataset (i.e., empirical data) to estimate BMI and waist circumference using regression coefficients from mixed models. This was done only for models that showed a significant association between movement behaviour composition and the given response variable. We used this approach to avoid potentially unrealistic estimations that could arise from using simulated compositions, some of which may never appear in real life. Given the presence of a few observations with outlying movement behaviour data, we performed regression diagnostics to assess the influence of these outliers on regression estimates. All outliers were retained in the final analytic dataset, as their influence on the regression estimates was negligible.

Third, the compositional mean of movement behaviour compositions associated with “healthy” BMI and waist circumference, alongside its bootstrap confidence interval (CI; derived as a component-wise projection from of the 95% confidence region, using Monte Carlo sampling), was calculated to estimate the optimal movement behaviour composition for healthy weight. We also synthesized the optimal movement behaviour compositions for “healthy” BMI and waist circumference by calculating their compositional mean. In addition, we used a similar procedure to estimate the optimal average acceleration and intensity gradient for healthy weight. For average acceleration and intensity gradient, we did not calculate isometric log-ratios, because they are not compositional data. For the same reason, instead of using the compositional mean to estimate their optimal values, we used the arithmetic mean.

The statistical analyses were performed in R version 4.4.0 (R Foundation for Statistical Computing, Vienna, Austria) using *compositions*, *nlme*, *robCompositions*, *robustbase*, and *VIM* packages.

## 3 Results

### 3.1 Characteristics of included studies and pooled sample

Our IPD meta-analysis included a pooled sample of 9,818 participants from four cross-sectional studies [46–49], one prospective cohort study [50], and six randomised controlled trials [51–56] (Table S1). The analytic sample was divided into four age groups, with the highest representation from older adults (51%), followed by children (21%), adolescents (13%), and adults (15%) (Table 1 and Figure S1). Most participants had medium to high SES, with those of low SES being the least represented across all age groups. This was particularly notable among adults, where only approximately 4% of participants had low SES.

In all included studies, data on movement behaviours were collected using wrist-worn accelerometers (Table S1). GENEAactiv accelerometers were used in five studies from Australia [51, 54], Chile [52], Spain [55], and the United Kingdom [48], with wear periods from 7 to 9 days. ActiGraph accelerometers, including GT9X, GT3X+, and wGT3X-BT models, were employed in studies conducted in Australia [53], Brazil [46], Czechia [49], New Zealand [47], and Spain [50, 56], with wear periods from 7 to 8 days. A total of 59,732 valid accelerometer-derived person-days were recorded. Among children, the mean movement behaviour composition was characterised by higher duration of sleep and lower duration of SB, compared with the remaining age groups (Table 1). The geometric mean of LPA was higher among children and adolescents, compared with adults and older adults. The lowest geometric mean for MVPA was found among adolescents. The highest average acceleration was found among children (79.2 ± 23.3 m*g*), followed by adolescents (45.4 ± 12.6 m*g*), adults (37.1 ± 11.2 m*g*), and older adults (30.9 ± 9.2 m*g*). The intensity gradient followed a similar age-related trend.

Regarding BMI, a “healthy” weight was most prevalent among adolescents (75.1%) and least prevalent among older adults (33.1%). The highest prevalence of overweight and obesity (65.9%) was found among older adults. Compared to prevalence of “healthy” BMI, a notably higher percentage of participants across all age groups had “healthy” waist circumference. The highest percentage of participants with “healthy” waist circumference was found among children (81.4%), followed by adults (54.2%), and older adults (50.3%). Waist circumference data were not available for adolescents.

### 3.2 Optimal composition of movement behaviours for healthy weight

Associations between the overall movement behaviour-composition and indicators of weight status were significant in all age groups (*p* ≤ 0.005 for all), except for waist circumference in children (*p* = 0.096; Table S2). The optimal composition of movement behaviours for healthy weight varied substantially across different age groups (Tables 2 and 3). Specifically, the optimal durations of sleep, SB, LPA, and MVPA ranged between 7.4–9.1, 9.9–12.6, 2.3–3.8, and 0.6–1.6 hours/day, respectively. For children, the optimal daily composition of movement behaviours for “healthy” BMI included 9.1 hours of sleep, 9.9 hours of SB, 3.8 hours of LPA, and 1.2 hours of MVPA. For adolescents, it included 7.4 hours of sleep, 12.6 hours of SB, 3.5 hours of LPA, and 0.6 hours of MVPA. The optimal daily composition of movement behaviours for healthy weight, calculated as a pooled estimate for “healthy” BMI and “healthy” waist circumference, consisted of 7.9 hours of sleep, 12.1 hours of SB, 2.4h of LPA, and 1.6 hours of MVPA for adults and 7.9 hours of sleep, 12.6 hours of SB, 2.3 hours of LPA, and 1.2 hours of MVPA for older adults. The results generally remained consistent after stratification by sex and SES (Tables S3–S6).

### 3.3 Optimal average acceleration and intensity gradient

When considering BMI as the outcome variable, the highest optimal average acceleration was found for children (80.5 m*g*; 95% CI: 80.5, 80.6), followed by adolescents (45.3 m*g*; 95% CI: 45.2, 45.3), adults (39.2 m*g*; 95% CI: 39.1, 39.3), and older adults (32.8 m*g*; 95% CI: 32.7, 32.9; Table 2). A similar age-related trend was found for the optimal intensity gradient, with the highest value found for children (−1.92; 95% CI: − 1.92, − 1.92), followed by adolescents (−2.24; 95% CI: − 2.25, − 2.24), adults (−2.47; 95% CI: − 2.47, − 2.47), and older adults (−2.64; 95% CI: − 2.64, − 2.64). The results for adults and older adults remained consistent in the models with waist circumference as the outcome variable (Table 3).

## 4 Discussion

There are several particularly important findings of this one-stage IPD meta-analysis. First, the movement behaviour composition consisting of sleep, SB, LPA, and MVPA is associated with BMI in all age groups and with waist circumference among adults and older adults. Second, the optimal movement behaviour composition for healthy weight is characterised by: 1.2–1.7 hours/day higher duration of sleep and 2.2–2.7 hours/day lower duration of SB among children, compared with the other age groups; more than 1 hour/day lower durations of LPA among adults and older adults, compared with children and adolescents; and 0.6–1.0 hours/day lower duration of MVPA among adolescents, compared with the remaining age groups. Third, for healthy weight, the highest overall activity (expressed using average acceleration) and the most uniform distribution of time across the activity intensity spectrum (expressed using intensity gradient) are required among children, followed by adolescents, adults, and older adults.

### 4.1 Optimal sleep duration

The optimal sleep durations for healthy weight among children, adolescents, adults, and older adults found in our study are similar to findings from Czechia [14, 18] and the Netherlands [17]. However, our estimate for children is nearly 2 hours/day lower than in a study from Australia [11]. The discrepancy between findings could be due to differences in accelerometer data processing methods, different distributions of movement behaviour compositions (reflected also in the estimated regions of empirical compositions), and differences in the associations between movement behaviour composition and weight-related outcomes.

The optimal sleep duration for healthy weight found in our study is consistent with sleep duration guidelines for children, adults, and older adults [57], but it is shorter than the recommended sleep duration for adolescents. This discrepancy is not surprising, because sleep duration guidelines also take into consideration other health outcomes, and the optimal sleep duration seems to vary across different outcomes [58]. From a public health perspective, the optimal sleep duration for healthy weight should be considered alongside optimal sleep durations for other important health outcomes.

### 4.2 Optimal duration of sedentary behaviour

Our analysis suggests that time in SB is the predominant part of optimal movement behaviour composition for healthy weight, representing more than 40% of the day among children and approximately half of the day in other age groups. Our estimate for optimal SB in children is 1.2 and 0.9 hours/day lower than in studies from Australia [11] and Czechia [14], respectively. Our estimates for adolescents and older adults are very similar to two studies from Czechia [14, 18], while our estimate for adults is 6 hours higher than in a study from the Netherlands [17]. The large discrepancy between the findings for adults is likely because in the previous study standing time was analysed as a separate part of the movement behaviour composition. It could be that in our study, some standing time was misclassified as SB and that in the previous study some SB time was misclassified as standing time.

According to the Canadian 24-Hour movement guidelines [59], adults and older adults should spend no more than 8 hours per day in SB. The optimal durations of SB for healthy weight in these age groups found in our study are substantially higher than the recommended amount of SB. However, as mentioned above, public health guidelines consider various health outcomes, which may explain the discrepancy from our findings. Given a lack of public health guidelines for the overall daily duration of SB among children and adolescents [60], there are no reference values that could be used for comparisons with our findings. To better understand our findings, we made comparisons with SB levels from large population-based studies using accelerometers. Our estimates are consistent with previous studies, which report SB ranges of 6 to 11 hours per day in children and adolescents and 8 to 12 hours per day in adults and older adults [61].

### 4.3 Optimal duration of light physical activity

The optimal durations of LPA for healthy weight among children, adolescents, and older adults found in our study are similar to findings from Czechia [14, 18]. However, our estimate for children is nearly three hours/day higher than in a study from Australia [11], while our estimate for adults is more than five hours/day lower than in a study from the Netherlands [17]. As mentioned earlier, the former could be explained by differences in data processing methods, empirical movement behaviour compositions, and associations between movement behaviours and weight-related outcomes, while the latter could be due to potential misclassification of standing time.

According to the Canadian 24-Hour movement guidelines, children, adolescents, adults, and older adults should spend several hours per day in LPA [59, 62]. However, no specific quantitative recommendation is provided. The findings of our study suggest that the optimal duration of LPA for healthy weight may vary across different stages of life. Our estimates align with previous studies that determined optimal movement behaviour composition for various physical and mental health outcomes. For example, Dumuid et al. [10] estimated that 1.7–5.1 hours/day of LPA are needed for optimal health and well-being among 11–12-years-old children, while Brakenridge et al. [17] found that 2.2 hours of LPA is required to achieve optimal cardiometabolic health in adults. Although a growing body of evidence has shown that LPA can confer health benefits [63], its relationship with weight status has not yet been fully explored and existing studies do not emphasise LPA in weight management [64–66]. Therefore, our findings can serve as an initial point for further research into establishing detailed recommendations for LPA in relation to weight status.

### 4.4 Optimal duration of moderate-to-vigorous physical activity

We found that time in MVPA is the smallest part of optimal movement behaviour composition for healthy weight, representing between 2.4% and 6.5% of the day in different age groups. However, this does not mean that MVPA is least important for healthy weight. Our estimates for children and adolescents are approximately 5–25 minutes/day higher than in previous studies from Australia [11] and Czechia [14]. Our estimate for adults is approximately 30 minutes/day lower than in a study from the Netherlands [17], while our estimate for older adults is almost the same as the estimate for the young-old from Czechia [18]. While the absolute differences between our estimates and the optimal durations of MVPA from previous studies may seem small, their relative magnitudes for children, adolescents, and adults are actually quite high. This could be due to methodological differences between the studies, particularly in terms of data processing.

Health benefits associated with moderate and vigorous physical activity have been the subject of extensive research in the past decades [67], leading to the development and refinement of quantitative public health guidelines for MVPA [68]. Our estimated optimal durations of MVPA for children, adults, and older adults are much higher than the recommended minimum duration of MVPA (i.e. 420 minutes/week for children and 150 minutes/week for adults and older adults). This could suggest that individuals with healthy weight tend to accumulate more MVPA than the minimum recommended levels. However, whether this finding reflects a causal dose-response relationship or selection effects warrants investigation in longitudinal and intervention studies. In contrast, our estimated optimal duration of MVPA for adolescents is significantly lower than the recommended minimum duration of MVPA (i.e. 420 minutes/week). This may indicate that adolescents with healthy weight tend to accumulate less MVPA than the current guidelines recommend. However, whether this reflects a genuinely lower MVPA requirement for weight maintenance among adolescents or potential residual confounding cannot be determined from these cross-sectional data. Moreover, this finding might be explained by the distinct characteristics of adolescence, including rapid growth and intense developmental processes, potentially attenuating the relationship between physical activity and weight status [69]. It is also important to emphasise that the current MVPA guidelines were developed mainly based on self-reported MVPA data [70] and analyses that did not take into account the compositional nature of time-use data [1], which may partially explain the differences from our estimates.

### 4.5 Optimal average acceleration and intensity gradient

This is the first study to determine the optimal average acceleration and intensity gradient for healthy weight; hence, no comparisons with previous studies were possible. Comparing optimal movement behaviour compositions determined in different studies may be challenging, primarily due to methodological issues related to the selection of acceleration thresholds for estimating SB and physical activity of different intensities, as well as the algorithms used for sleep detection. Average acceleration and intensity gradient should be considered for inclusion in future research as additional metrics, as they may facilitate making direct comparisons between studies [71].

Our findings suggests that the overall activity (estimated using average acceleration) required for healthy weight reduces with age. This is partially in agreement with the Canadian 24-Hour movement guidelines [59, 62], where the recommended weekly duration of MVPA is nearly three times higher for children and adolescents than for adults and older adults, while the recommended upper limit of screen time is one hour per day lower for children and adolescents than for adults and older adults. Our findings also suggest that the uniformity of distribution of time across the activity intensity spectrum (expressed using intensity gradient) required for healthy weight reduces with age. For healthy weight, younger individuals may require a more uniform distribution of time across the activity intensity spectrum, compared with adults and older adults. If confirmed in future studies, this novel finding could be considered in the development of future movement behaviour guidelines.

### 4.6 Implications for public health guidelines and practice

Current 24-Hour movement guidelines, such as the Canadian 24-Hour movement guidelines [59, 62], do not include quantitative recommendations for overall daily duration of SB among children and adolescents and for LPA in any age group. The findings of our study could be used to inform the development of such recommendations in future movement behaviour guidelines. Based on our findings, the future movement behaviour guidelines could also include specific recommendations for weight management. Expanded guidelines could be particularly useful for countries with a high prevalence of obesity. In addition to specifying the optimal composition of movement behaviours, new movement behaviour guidelines could also include additional recommendations on the distribution of activity throughout the day.

From a practical perspective, our findings could be encouraging for many adolescents who struggle to meet the MVPA guidelines but potentially discouraging for many adults and older adults whose MVPA duration is significantly below the optimum for healthy weight. Importantly, for health promotion purposes, it should be taken into account that the optimal duration of MVPA for adolescents found in our study may not be sufficient to achieve other important health benefits. It should also be noted that the relatively high optimal durations of MVPA for children, adults, and older adults may be challenging to attain. Therefore, our findings should be applied specifically for obesity prevention, rather than for general physical activity promotion purposes.

### 4.7 Strengths and limitations

Key strengths of our study include: 1) harmonised analysis of data collected using research-grade accelerometers in 11 studies from 7 countries, which enabled us to capture between-study and between-country variability; 2) application of compositional data analysis, which enabled us to adequately address compositional properties of time-use data; 3) the inclusion of average acceleration and intensity gradient in analyses, which could facilitate more robust comparisons with future studies; and 4) the use of both BMI and waist circumference to determine weight status [40].

There are also several limitations of this study. First, the cross-sectional study design precluded making causal inferences. Reverse causation (e.g. lower BMI enabling more MVPA) and residual confounding (particularly by dietary intake and maturation status which we could not harmonise across studies) may have impacted our findings. Second, the pooled sample of adolescents was formed from only three studies, potentially limiting the generalisability of findings for this age group. Third, there was generally a lack of data from low-income countries and a relatively small number of participants with low SES, potentially limiting the generalisability of our findings for all age groups. Fourth, the prevalence of overweight and obesity in our study was not perfectly aligned with the most recent population estimates [21, 22], also potentially affecting the external validity of our results. Finally, given that BMI and waist circumference were the only health outcomes considered in our study, we could not draw conclusions about the “optimum of optima”, that is, the optimal movement behaviour composition for overall health.

## 5 Conclusions

Movement behaviours are associated with weight-related outcomes in all age groups. The optimal movement-behaviour composition is characterised by higher duration of sleep and lower duration of SB among children, lower duration of MVPA among adolescents, and lower durations of LPA among adults and older adults, relative to the remaining age groups. The overall activity and uniformity of distribution of time across the activity intensity spectrum required for healthy weight seem to reduce with age. Our findings provide novel insights that could be used to optimise movement behaviours as part of weight management strategies across the lifespan, and to inform future movement behaviour guidelines. Future studies should analyse a broader range of outcomes to determine an optimal movement behaviour composition for health.

## Supplementary Files

This is a list of supplementary files associated with this preprint. Click to download.

- SupplementaryFiles.pdf
- SupplementaryFilesPRISMAIPD.pdf

## Figures and Tables

**Table 1:** Characteristics of study participants by age groups^a^

	Children		Adolescents		Adults		Older Adults	
	*n* = 2103		*n* = 1234		*n* = 1481		*n* = 5000	
	Mean	SD	Mean	SD	Mean	SD	Mean	SD
Age (years)	9.4	1.3	16.1	1.3	55.5	13.6	72.0	4.5
BMI^b^ (kg/m^2^)	18.1	3.3	21.9	3.8	26.8	5.0	27.0	4.3
BMI^b^ *z*-score	0.62	1.26	0.25	1.08	n/a	n/a	n/a	n/a
WC (cm)^c,d^	61.5	8.8	–	–	96.1	12.5	96.0	11.8
Accelerometry variables								
Wear time (min/day)	1423.8	31.5	1413.2	36.2	1439.2	21.3	1440.4	16.3
Average acceleration^e^ (mg)	79.2	23.3	45.4	12.6	37.1	11.2	30.9	9.2
Intensity gradient^f^	−1.93	0.14	−2.25	0.17	−2.50	0.20	−2.67	0.21
	*n*	%	*n*	%	*n*	%	*n*	%
Males	1027	48.8	592	48.0	892	60.2	3107	62.1
Socioeconomic status								
Low	336	16.0	139	11.3	53	3.6	1659	33.2
Medium	556	26.4	581	47.1	731	49.4	2125	42.5
High	604	28.7	476	38.5	549	37.0	1214	24.3
Missing	607	28.9	38	3.1	148	10.0	2	<0.1
Weight status according to BMI^g^								
Underweight	24	1.1	14	1.1	23	1.6	50	1.0
“Healthy” weight	1300	61.8	927	75.1	585	39.5	1657	33.1
Overweight and obesity	779	37.1	293	23.8	873	58.9	3293	65.9
Weight status according to WC^c,d,h^								
“Healthy”	722	81.4	–	–	572	54.2	2509	50.3
Above threshold	165	18.6	–	–	483	45.8	2484	49.7
	*g* ^i^	var^j^	*g* ^i^	var^j^	*g* ^i^	var^j^	*g* ^i^	var^j^
Movement behaviors (min/day)								
Sleep	545.2	35.3	439.7	38.8	474.1	40.4	468.2	40.1
Sedentary behaviour	591.8	49.7	751.9	44.5	726.9	49.9	758.6	46.4
Light physical activity	229.0	38.3	213.4	35.3	143.7	40.0	141.4	37.3
MVPA^k^	74.0	76.7	35.0	81.4	95.3	69.7	71.8	76.2

a Children (5–12 yrs of age), adolescents (13–17 yrs of age), adults (18–64 yrs of age), and older adults (65 + yrs of age)

b Body mass index

c Waist circumference

d There were missing data for this variable among children (*n* = 1216), adults (*n* = 179), and older adults (*n* = 7). Data were not available for adolescents.

e Average acceleration across the 24-hour day, expressed in the units of Earth’s gravitational acceleration

f Intensity gradient, with values closer to zero reflecting a more even distribution of time across the activity intensity spectrum.

g Classified according to the World Health Organization.

h The 85th sex- and age-specific percentile was used as a threshold for children [41]. Sex- and BMI-specific thresholds were used for adults and older adults [43].

i Robust compositional mean

j The part of total variance related to a given movement behaviour

k Moderate-to-vigorous physical activity

**Table 2:** Optimal movement behaviour composition, average acceleration, and intensity gradient for “healthy” BMI^a^ in different age groups^b^

	Children		Adolescents		Adults		Older adults	
	*n* = 2103		*n*=1234		*n*=1481		*n* = 5000	
	x*^c^	95% CI^d^	x*^c^	95% CI^d^	x*^c^	95% CI^d^	x*^c^	95% CI^d^
Sleep (min/day)	545	545, 546	441	441, 442	474	474, 475	476	475, 476
SB^e^ (min/day)	594	593, 594	754	753, 754	720	719, 721	746	745, 747
LPA^f^ (min/day)	227	227, 228	211	211, 212	152	151, 152	143	142, 143
MVPA^g^ (min/day)	74	73, 74	34	34, 34	94	93, 94	76	75, 76
Average acceleration^h^ (mg)	80.5	80.5, 80.6	45.3	45.2, 45.3	39.2	39.1, 39.3	32.8	32.7, 32.9
Intensity gradient^i^	−1.92	−1.92, −1.92	−2.24	−2.25, −2.24	−2.47	−2.47, −2.47	−2.64	−2.64, −2.64

a Body mass index between 18.5 and 24.9 kg/m^2^

b Children (5–12 yrs of age), adolescents (13–17 yrs of age), adults (18–64 yrs of age), and older adults (65 + yrs of age)

c Optimal value

d 95% bootstrap confidence interval for x*

e Sedentary behaviour

f Light physical activity

g Moderate-to-vigorous physical activity

h Average acceleration across the 24-hour day, expressed in the units of Earth’s gravitational acceleration

i Intensity gradient, with values closer to zero reflecting a more even distribution of time across the activity intensity spectrum

**Table 3:** Optimal movement behaviour composition, average acceleration, and intensity gradient for “healthy” WC^a^ in different age groups^b^

	Adults		Older adults	
	n = 1481		*n* = 5000	
	x* ^c^	95% CI^d^	x* ^c^	95% CI^d^
Sleep (min/day)	474	473, 474	473	472, 473
SB^e^ (min/day)	737	736, 738	762	761, 762
LPA^f^ (min/day)	138	137, 138	137	137, 137
MVPA^g^ (min/day)	92	91, 92	69	68, 69
Average acceleration^h^ (mg)	35.6	35.5, 35.7	31.1	31.1, 31.2
Intensity gradient^i^	−2.53	−2.53, −2.53	−2.67	−2.67, −2.67

a Waist circumference, sex- and body mass index-specific thresholds were used [43].

b Adults (18–64 yrs of age) and older adults (65 + yrs of age)

c Optimal value

d 95% bootstrap confidence interval for x*

e Sedentary behaviour

f Light physical activity

g Moderate-to-vigorous physical activity

h Average acceleration across the 24-hour day, expressed in the units of Earth’s gravitational acceleration

i Intensity gradient, with values closer to zero reflecting a more even distribution of time across the activity intensity spectrum (i.e. more favourable activity profile)

## Data Availability

The study protocol and analytic code are available on request from the corresponding author. Data used in this article were obtained, with permission, from original data custodians. These de-identified data may be available to researchers who provide a methodologically sound proposal. Requests for access to data can be emailed to Dr Hartwig (timothy.hartwig@acu.edu.au). Data share agreements may need to be signed.

## Key points

- The optimal daily movement behaviour composition is characterised by longer sleep duration and shorter duration of sedentary behaviour among children, shorter duration of moderate-to-vigorous physical activity among adolescents, and shorter durations of light physical activity among adults and older adults, when compared to the remaining age groups.
- The overall activity level (estimated using average acceleration) and uniformity of distribution of time across the activity intensity spectrum (estimated using intensity gradient) required for healthy weight reduce with age.

## Code availability

The script used for harmonising accelerometer data is publicly available at https://motivation-and-behaviour.github.io/sleepIPD
